# Comprehensive treatment of Cronkhite-Canada syndrome: A case report and literature review

**DOI:** 10.1097/MD.0000000000032714

**Published:** 2023-02-10

**Authors:** Hailong Hu, Yating Wu, Yaqin Zhang, Li Zhang, Jianfa Zhang, Rui Zhang

**Affiliations:** a Department of General Surgery, Huanghe Sanmenxia Hospital, Sanmenxia, China; b First Clinical Medical College, Nanjing Medical University, Nanjing, China.

**Keywords:** case report, Cronkhite-Canada syndrome, gastrointestinal polyps, literature review, traditional Chinese medicine

## Abstract

Cronkhite-Canada syndrome (CCS) is currently considered to be a non-hereditary disease, which is relatively rare clinically. It is also known as polyposis hyperpigmentation alopecia nail dystrophy syndrome, it is a syndrome characterized by gastrointestinal polyposis and ectodermal changes, the main manifestations are gastrointestinal symptoms, skin pigmentation, alopecia, and hypothyroidism.

**Case presentation:**

In this paper, the clinical characteristics, diagnosis and treatment of a case of CCS admitted to Huanghe Sanmenxia Hospital were analyzed. In the course of treatment, traditional Chinese medicine was used, but no hormone, and the patient’s clinical symptoms were greatly relieved.

**Conclusions:**

CCS is rare, there is no specific treatment, and traditional Chinese medicine may can greatly relieve the clinical symptoms of patients. However, it’s still having to be verified by a large sample, multi-center, long-term treatment follow-up studies.

## 1. Introduction

Cronkhite-Canada syndrome (CCS) was first reported in 1955,^[1]^ Only about 500 cases have been reported worldwide, of which approximately 75% were from Japan, with the largest cohort available was from Japan,^[2]^ The average age of onset was 59 years, with more than 80% of the patients were over 50 years old at diagnosis^[3]^ The male to female ratio of cases was 1.5 to 2:1.^[4]^

The main clinical manifestations of CCS include: The clinical manifestations of gastrointestinal polyps such as diarrhea, abdominal pain, nausea, vomiting, weight loss, etc, can lead to gastrointestinal bleeding and intussusception and other serious complications.^[3]^ Ectodermal symptoms such as alopecia, nail dystrophy (atrophy, thickening, or abscission) and pigmentation, over 60% of patients combined with hypogestation.^[5]^ Pigmentation in CCS can be diffuse or focal, often involving the limbs, face, neck, and occasionally the lips.^[6]^

CCS often affects both the upper and lower digestive tract, but usually does not affect the esophagus^[7]^; Multiple gastrointestinal polyps are usually sessile and can appear as overlapping polyps or as diffuse mucosal thickening or atrophy.^[3]^ The histopathological features of classical CCS polyps include lamina propria edema, cystic dilation of glands, mononuclear cell infiltration, etc. Histologically, they are classified as hamartoma polyps, which overlap with juvenile polyps to some degree,^[8]^ However, the mucosa with normal appearance between the lesions may also have histological abnormalities.^[6]^

There is no specific treatment for CCS because the pathogenesis is unknown. In recent years, glucocorticoid-based immunosuppressive therapy has gradually become the mainstream treatment choice. However, there is no relevant report on traditional Chinese medicine in the treatment of this disease. In this paper, traditional Chinese medicine therapy was combined with nutritional support therapy, and the patient’s clinical symptoms were significantly alleviated.

## 2. Case presentation

A 47-year-old male patient was admitted to Sanmenxia Hospital due to inappetence and fatigue for 1 year on February 24, 2022. One year ago, the patient developed inappetence and fatigue without obvious inducement, and was not treated at that time. Then the above symptoms gradually worsened, accompanied by abdominal distension, diarrhea, loss of hair and nails, skin pigmentation on hands and feet, brown rash on face, and weight loss of 4 kg. His personal and past histories were unremarkable.

**Figure 1.**
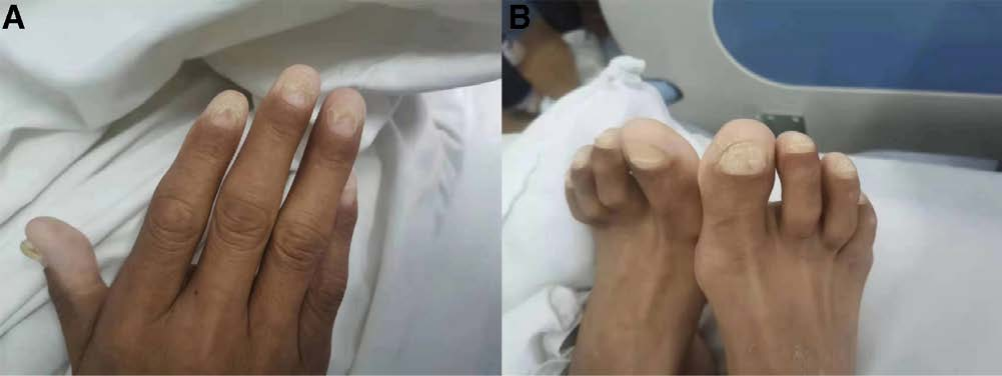
The nail layer of the patient’s finger (toe) has come off. Local pigmentation of fingers (toes) (A fingers, B toes).

On his physical examination at admission: blood pressure and heart rate were 87/58 mm Hg and 76 beats/minutes, And the respiratory rate was 19 breathes/minutes. Slightly worse spirit; The hair was sparse, brown pigmentation was observed in many parts of the limbs, and the nail layer of the fingers (toes) fell off (Fig. 1). Heart, lung, and abdominal examinations were negative.

**Figure 2.**
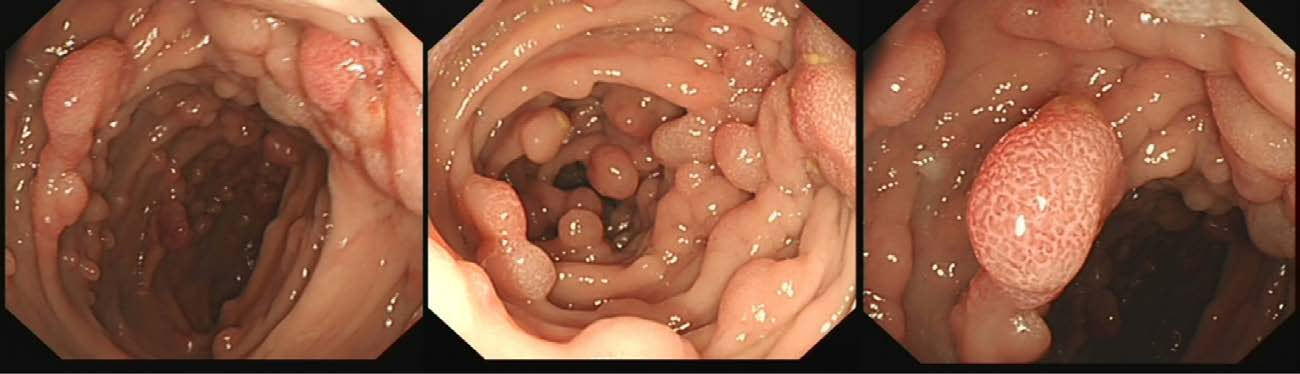
Multiple polyps formed in the colon.

**Figure 3.**
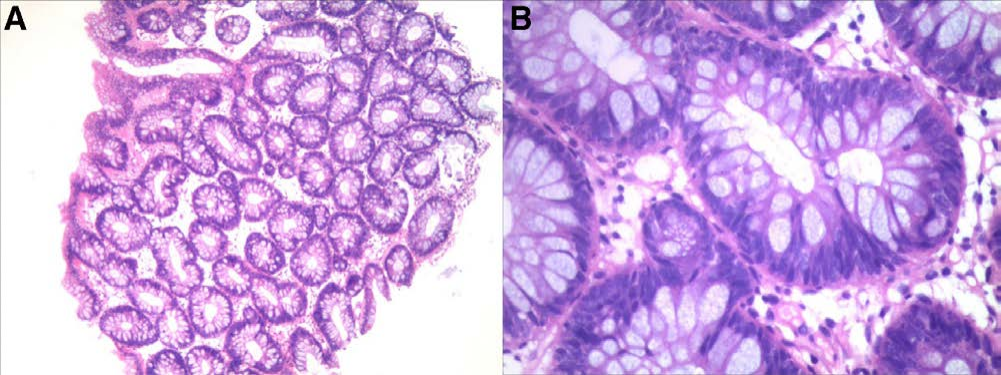
Pathology of sigmoid colon polyps (A HE × 40, B HE × 200).

Examination before treatment: Anemia, poor nutritional status, abnormal immune tests, and occult blood test (+). The results are shown in Table 1 (before). Colonoscopy showed that the ileocecal region was lip-shaped, scattered polypoid protrusions could be seen in the rectum, and a large number of polypoid protrusions of different sizes could be seen in the whole colon, the surface was congested and sessile, the maximum diameter was about 2.0*2.5 cm, and the surface glands were dilated (Fig. 2). Biopsy tissue was taken from the sigmoid colon, and the pathological examination results showed tubular adenoma (sigmoid colon) (Fig. 3). Abdominal CT showed thickened antral pylorus wall, a small amount of peritoneal fluid, slightly enlarged retroperitoneal and abdominal lymph nodes. Urine routine, renal function, thyroid function, tumor markers, chest X-ray, cardiac color Doppler ultrasound, anti-neutrophil cytoplasmic antibodies antibody spectrum, and C-reactive protein were normal.

**Table 1:** Laboratory findings before and after treatment.

Laboratory variable		Before	After	Normal range
Blood	White blood cell(*1012/L)	6.86	7.28	3.5–9.5
	Red blood cell (*1012/L)	3.83	4.17	4.3–5.8
Hemoglobin (g/L)		95	101	130–175
Hematocrit (%)		32	33.9	40.0–50.0
Total protein (g/L)		44.1	44.9	68.0–85.0
Albumin (g/L)		26.1	27.2	40.0–55.0
Globulin (g/L)		18	17.7	20.0–40.0
Immunoglobulin G (mg/dL)		428	672	700–1600
Immunoglobulin E (IU/mL)		933	N/A	0–100
Complement C3 (mg/dL)		68.4	86	79–152
Complement C1q (mg/L)		125.2	N/A	159–233
Anti-PM-Sc1		+	+	-
Stool	Occult blood test	+	-	-
	White blood cells	-	-	-
	Parasitological	-	-	-

A consultation team (consisting of specialists from our hospital’s Gastroenterology, General surgery, Hematology, Rheumatology and immunology department) was involved in the diagnosis of the case. Based on the patient’s medical history and examination results, The clinical diagnosis: CCS; Iron deficiency anemia.

First, we suggested the patient to use hormone, but the patient refused for fear of side effects and requested traditional Chinese medicine treatment. After discussion with the Chinese medicine doctor, the following treatment plan was formulated. Proton pump inhibitor (PPI), fat-soluble vitamins, water-soluble vitamins, a variety of trace elements, fat emulsion, amino acids, intravenous nutrition, and traditional Chinese medicine (Poria 30g; processed licorice 10g; atractylodes 30g; costusroot 10g; papaya 10g; Amomum 10g; big belly skin 12g; dried ginger 10g; magnolia officinalis 10g; decoction, once a day) were given. Iron sulfate and folic acid were given to correct anemia.

After a week of medication, the patient’s diet increased, his mental condition improved, and his diarrhea was relieved. Reexamination after treatment: anemia and nutritional indicators were improved, the immune indexes tended to be normal. Results are shown in Table 1 (after). The above treatments were continued with the addition of traditional Chinese medicine (cuttlebone 15 g; cherry blossoms 15 g; dodder 15 g; chicken’s gizzard-membrane 30 g; hawthorn 20 g; medicated leaven 20 g; colored malt 20 g; Earthworm 10 g).

Five days after the second treatment, the patient’s discomfort basically disappeared, and he asked to continue outpatient follow-up. To date, the patient has been followed up for 4 months, and no clinical manifestations have been observed, the patient was satisfied with the treatment, at the same time, we suggested that the patient complete the colonoscopy again, but the patient refused on the grounds of no discomfort.

## 3. Discussion and conclusions

The incidence of CCS is about 1/1.000.000, which is a rare disease. It is a syndrome characterized by gastrointestinal polyposis and ectodermal changes, and its etiology and pathogenesis are currently unclear.^[9]^ The clinical manifestations of this disease are diverse, and the diagnosis must have both ectodermal changes and multiple gastrointestinal polyps. The prognosis is bad, and most patients die within a short period after the onset of symptoms.

The etiology and pathogenesis of CCS are still unclear, but it involves genetic abnormalities, abnormal proliferation and differentiation of intestinal epithelial cells, immune system abnormalities and stress.^[10]^ At present, it is generally considered to be a chronic inflammatory disease associated with autoimmune mechanisms, and the current evidence mainly includes ANA positivity,^[11]^ the level of IgG4 in the blood circulation was increased,^[12]^ polyps infiltrated with IgG4-positive plasma cells,^[13]^ and an overall good clinical response to immunosuppressive therapy.^[2]^ In addition. Multiple studies have found that Helicobacter pylori infection is associated with CCS,^[14]^ CCS was relieved after anti-H pylori treatment.^[15]^ Another study suggests that mental stress may lead to local inflammation of the gastrointestinal mucosa,^[16]^ this may be one of the potential mechanisms of mental factors causing this disease.

At present, the research tends to believe that CCS is a non-genetic disease, and there are no such cases in the patient’s family in this report. However, a case report from India showed that the father and son were successively diagnosed with CCS.^[17]^ A transcriptome analysis study found that the upward regulation of inhibin subunit beta A gene upregulation may be related to the development of inflammation and multiple malignant polyps in CCS patients.^[18]^ Therefore, the role of genetic factors in the occurrence and development of CCS needs to be verified by more genetic and epidemiological studies.

Gastrointestinal symptoms are the main manifestations of CCS, including abdominal pain and diarrhea, which can also be accompanied by frequent nausea and acid regurgitation, anorexia, abnormal taste, or fatigue. Some patients may have skin pigmentation on the back, hands and feet, some patients have nail dystrophy involving 1 or more fingers (toes), and some patients have alopecia. A small number of patients can be accompanied by other autoimmune diseases, such as membranous nephropathy, autoimmune pancreatitis, systemic lupus erythematosus, vitiligo, rheumatoid arthritis, scleroderma, hypothyroidism, and adult-onset Still’s disease.^[19,20]^ Due to hair follicles of the scalp show no evidence of histological alterations or inflammatory lesions, Da Porto A et al assumed that the ectodermal tissue alterations may be caused by malabsorption of micronutrients and minerals, such as zinc and iron.^[21]^ In this case, the patient’s clinical symptoms improved after 3 months of application of trace elements. CCS is characterized by the presence of diffuse gastrointestinal polyps, and multiple diffuse gastrointestinal polyps often involve the entire gastrointestinal tract outside the esophagus,^[22]^ It is most commonly found in the stomach and colon, followed by the small intestine and rectum. Most of the gastric polyps and colonic polyps are sessile, mucosal congestion, and rare punctate bleeding. Gastric polyps are smaller and more confluent than colonic polyps.^[2]^ There is no typical pathological type of CCS, and 4 histological types of polyps have been found in patients with CCS: hyperplastic, adenomatous, juvenile and inflammatory.^[23]^ Approximately 12.5% of polyps have been reported to undergo malignant transformation, emphasizing the need to closely monitor these patients.^[24]^

The diagnosis of CCS is based on medical history, physical examination, endoscopic examination of gastrointestinal polyps and histopathology. Digestive endoscopy is the most direct examination method for this disease. Because there is considerable overlap between the endoscopic and histological characteristics of CCS polyps and other polyposis syndromes, the CCS diagnosis is based on clinicopathological evidence, not only histological evidence.^[25]^ The main diagnostic difficulty is to distinguish CCS polyps from juvenile polyposis syndrome. Compared with juvenile polyposis syndrome, CCS polyps have fewer pedicles, inflammatory cell infiltration and edema in the lamina propria, and polyps have mucous abnormalities.^[22]^ In addition, juvenile polyposis syndrome does not have epidermal features is also a differential point.^[26]^ Despite the high rate of coincidence between CCS with gastrointestinal and colorectal cancers, it is unclear whether CCS is precancerous or whether it is associated with progression of conventional adenoma-carcinoma sequences.^[20]^

At present, there is no effective treatment for CCS. The common treatment methods include conservative medical and surgical treatment. At present, glucocorticoid-based non-suppressive therapy is the mainstream treatment. The average recovery time for diarrhea, taste abnormalities and ectodermal changes was 51 days, 84 days, and 9 days. The average regression time of gastric and colonic polyps was 248 days and 238 days,^[2]^ but it is not clear whether this transform will alter the natural history of the disease.^[20]^ Besides PPI and cromolyn sodium have been used, particularly in patients with eosinophilia on biopsy.^[27]^ Active nutritional support, such as a high-protein diet and fluid and electrolyte supplementation, is also an important pillar of CCS treatment.^[20]^ Surgery is only used to treat complications such as severe protein-losing enteropathy, obstruction, prolapse, persistent hematochezia or hematemesis, and malignant transformation, but rarely to eliminate gastrointestinal polyps.^[28]^

In the treatment of this case, traditional Chinese medicine therapy was used, combined with the preparation of PPI and nutritional support treatment, and the patient’s clinical symptoms were greatly alleviated. From the point of view of traditional Chinese medicine, the main symptoms of the patient belong to the deficiency of spleen Yang, stagnation of fluid-dampness, so the application of Shipi Drink treatment, which has the role of invigorating Qi and strengthening the spleen, promoting Qi circulation to induce diuresis, checking diarrhea, relieving gastric hyperacidity to alleviate stomachache. However, the modern pharmacological studies have shown that polysaccharide and triterpenoids in Poria can regulate the function of immune organs of spleen and thymus. In addition, processed licorice, similar with adrenocorticotropic hormone, can adjust the immune system. Atractylodes can increase the phagocytic function of reticuloendothelial system, especially in patients with leukopenia. Atractylodes can also increase the leukocyte, promote cellular immune function, and significantly increase the level of IgG.^[29,30]^ Chicken’s gizzard-membrane can promote gastrointestinal motility and improve diet, and there are a lot of trace elements (iron, magnesium, copper, zinc, manganese, etc) in chicken inner gold and dodder. Each of these components addresses different possible causes of CSS.^[31,32]^ However, the specific mechanism of action and the interaction of traditional Chinese medicine still needs further study.

In conclusion, CCS is rare, the pathogenesis is unknown, and there is no unified diagnosis and treatment standard. On the basis of summarizing the clinical characteristics, pathogenesis, diagnosis and treatment methods of the disease, this case report has achieved good clinical effect by applying traditional Chinese medicine treatment scheme. The diagnosis and treatment experience of this patient has increased the confidence in the treatment of patients with CCS. It is hoped that more case data can be collected and analyzed to provide more possibilities for the treatment of CCS.

## Author contributions

**Data curation:** Yaqin Zhang, Jianfa Zhang.

**Investigation:** Hailong Hu, Yating Wu, Li Zhang, Jianfa Zhang.

**Project administration:** Li Zhang.

**Resources:** Jianfa Zhang.

**Supervision:** Hailong Hu, Jianfa Zhang.

**Writing – original draft:** Yaqin Zhang, Rui Zhang.

**Writing – review & editing:** Rui Zhang.
